# Monitoring CD8a^+^ T Cell Responses to Radiotherapy and CTLA-4 Blockade Using [^64^Cu]NOTA-CD8a PET Imaging

**DOI:** 10.1007/s11307-020-01481-0

**Published:** 2020-02-21

**Authors:** Lotte K. Kristensen, Camilla Christensen, Maria Z. Alfsen, Sigrid Cold, Carsten H. Nielsen, Andreas Kjaer

**Affiliations:** 1 Minerva Imaging, Copenhagen, Denmark; 2 Department of Clinical Physiology, Nuclear Medicine & PET and Cluster for Molecular Imaging, Department of Biomedical Sciences, Rigshospitalet and University of Copenhagen, Copenhagen, Denmark

**Keywords:** Molecular imaging, Positron emission tomography (PET), Immune cell imaging, Immunotherapy, Immune checkpoint inhibition, Response monitoring, Tumor-infiltrating lymphocytes, T cells, Cytotoxic T cells, CD8

## Abstract

**Purpose:**

Current response assessment systems for cancer patients receiving immunotherapy are limited. This is due to the associated inflammatory response that may confound the conventional morphological response evaluation criteria in solid tumors and metabolic positron emission tomography (PET) response criteria in solid. Recently, novel PET imaging techniques using radiolabeled antibodies and fragments have emerged as a particularly sensitive and specific modality for quantitative tracking of immune cell dynamics. Therefore, we sought to investigate the utility of Cu-64 labeled F(ab)′2 fragments for *in vivo* detection of CD8a^+^ T cells as a prognostic imaging biomarker of response to immunotherapy in an immunocompetent mouse model of colorectal cancer.

**Procedures:**

[^64^Cu]NOTA-CD8a was produced by enzymatic digestion of rat-anti-mouse CD8a antibody (clone YTS169.4), purified yielding isolated CD8a-F(ab)′2 fragments and randomly conjugated with the 2-*S*-(isothiocyanatbenzyl)-1,4,7-triazacyclononane-1,4,7-triacetic acid (*p*-SCN-Bn-NOTA) chelator. NOTA-CD8a was radiolabeled with Cu-64 and injected into CT26 tumor-bearing mice for longitudinal assessment. To investigate the value of [^64^Cu]NOTA-CD8a PET imaging for assessment of treatment response, CT26 tumor-bearing mice were subjected to external radiation therapy (XRT) in combination with anti-CTLA-4 therapy. Imaging data was supported by flow cytometry and immunohistochemistry (IHC).

**Results:**

Combination treatment with XRT and anti-CTLA-4 effectively inhibited tumor growth until day 22 post-therapy initiation (*p* = 0.0025) and increased the overall survival of mice compared to control (*p* = 0.0017). The [^64^Cu]NOTA-CD8a tumor-to-heart ratio was increased in XRT + anti-CTLA-4-treated mice on day 8 after initiation of therapy (*p* = 0.0246). Flow cytometry and IHC confirmed the increase in tumor-infiltrating CD8a^+^ cells in XRT + anti-CTLA-4-treated mice. Furthermore, [^64^Cu]NOTA-CD8a PET imaging distinguished responders and non-responders prior to treatment-induced changes in tumor volume among mice.

**Conclusion:**

In the present study, we demonstrated that [^64^Cu]NOTA-CD8a was able to detect treatment-induced changes in CD8a^+^ infiltration in murine CT26 colon tumors following a common preclinical combination treatment protocol. Overall, [^64^Cu]NOTA-CD8a exhibited good prognostic and predictive value. We suggest that [^64^Cu]NOTA-CD8a PET imaging can be used as an early biomarker of response to therapy in preclinical models.

## Introduction

The remarkable, yet variable, responses to cancer immunotherapy highlight the pressing need for biomarkers that can assist in patient selection and evaluation of treatment response. Alongside the diverse efficacies reported among patients and different tumor types comes the potential development of severe autoimmune-like adverse events that could be spared if appropriate treatment was selected at an early stage [1–3].

The clinical need is not limited to suitable, predictive biomarkers, but a strong urgency also exists for reproducible and standardized methods to systematically monitor the expression and dynamics of these [4]. Routine clinical biomarkers should preferably be assessed in a minimally invasive way, and ideally, longitudinally. Standard Response Evaluation Criteria in Solid Tumors (RECIST) are considered inadequate in discriminating responders from non-responders in patients receiving immune checkpoint blockade. This is due to atypical response patterns such as the associated inflammatory response that can cause an increase in tumor volume, *i.e.*, pseudo-progression [5–7]. To account for this, immune-related response assessment systems (irRC, irRECIST, iRECIST) for evaluation of response to immunotherapy have been implemented [8]. However, these modified criteria are merely related to tumor size and limited for early assessment of treatment response [5, 9]. While providing useful information regarding metabolic status, positron emission tomography (PET) Response Criteria in Solid Tumors (PERCIST) are correspondingly insufficient to identify patients with a favorable response to immunotherapy due to the inflammatory flare causing increased uptake by the metabolically active immune cells, *i.e.*, also a pseudo-progression [10]. Consequently, much research has been committed to develop molecular imaging techniques to evaluate and monitor immune status to spare patients from expensive and ineffective treatments with potential severe side effects.

CD8^+^ cytotoxic lymphocytes play an essential role in the anti-tumor immune response and their activation and infiltration in tumors are considered feasible candidates for assessment of immunotherapeutic response. This is owing to the fact that clinical responses to PD-1/PD-L1 immune checkpoint inhibitors arise most often in patients with high preexisting numbers and/or infiltration of CD8^+^ cells during therapy [11–14]. Molecular imaging probes targeting CD8^+^ have indeed been pursued in preclinical studies primarily utilizing engineered antibody fragments [15–18] and successfully demonstrated the value of CD8^+^ PET imaging for evaluation of immunotherapeutic response [16–18].

In the current study, we developed a Cu-64 labeled (*t*_1/2_ = 12.7 h) F(ab)′2 fragment targeting murine CD8a^+^ for non-invasive *in vivo* detection and quantification CD8^+^ cytotoxic lymphocytes. We sought to investigate the utility of [^64^Cu]NOTA-CD8a as a prognostic imaging biomarker of therapeutic response in an immunocompetent mouse model of colon adenocarcinoma. We used a combination therapy approach as these are currently widely investigated clinically due to the inherent differential response rates observed with monotherapy immune checkpoint inhibition [19–21]. Radiotherapy combined with immune checkpoint inhibition of CTLA-4 effectively inhibited tumor growth, increased the overall survival of CT26 tumor-bearing mice, and [^64^Cu]NOTA-CD8a PET identified the responding mice before tumor growth inhibition was evident.

## Materials and Methods

### Animal Model

CT26.WT murine colon carcinoma cells were acquired from ATCC (CRL-2638, LGC Standards) and tested negative for murine pathogens. Cells were cultured in RPMI-1640 + Glutamax™ medium supplemented with 10 % FCS and 1 % penicillin-streptomycin at 37 °C and 5 % CO_2_ according to standard procedures. Once in their exponential growth phase, cells were harvested and resuspended at 0.3 × 10^6^ cells/ml in PBS. One hundred microliters of cell suspension (300,000 cells/tumor) was injected subcutaneously (1 tumor/mouse) above the hind limbs in female BALB/c mice (approximately 6 weeks of age, Janvier Labs).

Tumors used for longitudinal imaging and biodistribution studies (*N* = 4) were grown until ~ 300 mm^3^ prior to injection with [^64^Cu]NOTA-CD8a. Tumors for the efficacy study and *ex vivo* analysis (*N* = 48) were grown for 13 days (~ 100–150 mm^3^) prior to treatment.

### Radiation Therapy, Immune Checkpoint Inhibition, and Tumor Monitoring

Mice were randomized into three treatment groups: non-treated controls, external radiation therapy (XRT), and a combination therapy group receiving XRT and anti-CTLA-4 (*N* = 16/group). Radiation therapy was delivered as fractionated doses with a dose rate of 1 Gy/min (320 kV, 12.5 mA, 120 s) using a small animal irradiator (XRAD-320, PXi) for three consecutive days (days 0, 1, and 2). Mice were placed in a restrainer allowing total fixation of the leg and the body was covered by lead shielding so only the tumor-bearing leg was irradiated. After the last radiation dose, mice from the combination therapy group received three intraperitoneal doses of 10 mg/kg anti-mouse CTLA-4 (9H10, #BE0131, BioXcell) on days 2, 4, and 6. Tumors were monitored by caliper measurements three times weekly until first measurement above 1500 mm^3^ or until end of study (day 92).

Six mice from each treatment group were euthanized on day 8 following therapy initiation. Tumors and spleens were excised, halved, and weighed. One half was placed in 4 % paraformaldehyde solution for 24 h followed by exchange to 70 % ethanol and paraffin embedding and was stored until immunohistochemical analysis. The other half was placed in MACS Tissue Storage Solution (#130-100-008, Miltenyi Biotec) on ice and immediately processed into single cell suspensions for flow cytometric analysis.

### Synthesis of [^64^Cu]NOTA-CD8a

R-anti-mouse CD8a clone YTS169.4 (#BE0117, BioXcell) was digested in the hinge region into F(ab)′2 and Fc fragments using FabRICATOR enzyme (#A0-FR1-050, Genovis). CD8a antibody in PBS was incubated with FabRICATOR for 2.5 h at 37 °C under continuous rotation. The crude antibody-enzyme mixture was purified by preparative HPLC (Yarra-2000 SEC column, 0.1 M phosphate buffer, 1 ml/min) yielding isolated F(ab)′2 and Fc fragments. CD8a-F(ab)′2 fragments were randomly conjugated to the 2-*S*-(isothiocyanatbenzyl)-1,4,7-triazacyclononane-1,4,7-triacetic acid (*p*-SCN-Bn-NOTA, Macrocyclics) chelator by incubating ~ 1 mg purified CD8a-F(ab)′2 fragments with 10× molar excess *p*-SCN-Bn-NOTA dissolved in DMSO in 0.1 M NaHCO_3_ (24 h, 37 **°**C, pH = 9.0). Following incubation, the mixture was purified on a PD-10 desalting column (GE Healthcare) into PBS. NOTA-CD8a-F(ab)′2 was aliquoted into 100 μg fractions and stored at − 80 °C until radiolabeling. Degree of labeling (DOL) of NOTA-CD8a-F(ab)′2 was determined by reverse-phase high performance-liquid-chromatography (HPLC) on a XBridge Protein BEH C4 Column 300 Å, 3.5 μm, 4.6 mm × 150 mm (Waters).

Two gigabecquerel [^64^Cu]CuCl_2_ (DTU Nutech, DTU) was dissolved in TraceSelect water (Merck Millipore) to a final concentration of 1 GBq/ml. NOTA-CD8a-F(ab)′2 (100 μg, 120 μl in PBS) was incubated with 250 MBq [^64^Cu]Cl_2_ in 0.1 M NaoAc buffer pH = 5.5 with 5 mg/ml gentisic acid (15 min, 37 °C). The reaction was quenched with 5 μl 10 mM EDTA followed by PD-10 purification into PBS. The radiochemical yield and purity at end-of-synthesis (EOS) were determined by size-exclusion-chromatography–HPLC (SEC-HPLC) using an isocratic method with 0.1 M phosphate buffer pH = 7 as mobile phase and a flow rate of 1 ml/min.

### SDS-PAGE

Full length CD8a^+^ antibody, reduced full length CD8a^+^ antibody, and [^64^Cu]NOTA-CD8a were mixed with NuPAGE LDS sample buffer (#NP0007, Invitrogen) and denatured for 10 min at 70 °C and loaded onto Bolt™ 4–12 % Bis-Tris gels (#NW04120, Invitrogen). Electrophoresis was run on the Mini Gel Tank system (Life Technologies) at 200 V constant voltage in NuPAGE MES SDS running buffer (#NP0002, Invitrogen). The gel was analyzed for radioactive content by exposure to Multisensitive Phosphor Screens and imaged using the Amersham Typhoon Imaging system (GE Healthcare). Subsequently, the gel was fixed and stained with Coomassie brilliant blue R-250 (#1610436, Bio-Rad).

### Small Animal PET/CT Imaging

*In vivo* PET/CT imaging was conducted in a subgroup of mice for longitudinal imaging (*N* = 4) and in mice from the efficacy study (*N* = 30). [^64^Cu]NOTA-CD8a (2.86 ± 0.07 MBq, 1.37 ± 0.03 μg) was injected together with a 30 μg co-dose of unlabeled CD8a-F(ab)′2 in PBS (~ 200 μl) intravenously *via* the tail vein as a single bolus dose. PET/CT imaging was conducted 1, 3, 6, 24, and 48 h after injection in mice for longitudinal assessment. Mice in the efficacy study were subjected to PET/CT imaging 24 h after injection on day 8 following therapy initiation. Imaging was performed on a Multimodality PET/CT scanner (Siemens) and mice were anesthetized prior to and during the imaging session in 3–4 % sevoflurane in 80 % N_2_ and 20 % O_2_. PET data were acquired in list mode with acquisition times of 300, 300, 300, 600, and 1200 s for the 1, 3, 6, 24, and 48 h timepoint, respectively. Static PET data were reconstructed using a 3D maximum *a posteriori* algorithm with CT-based attenuation correction.

### Image Analysis and Stratification

Image analysis was performed by drawing CT-based regions of interest (ROIs) of the tumor, whole heart, liver, kidney, and muscle using Inveon Software (Siemens). ROIs were drawn over the spleen by PET-based thresholding. The uptake of [^64^Cu]NOTA-CD8a was quantified as % injected dose per gram (%ID/g) tissue assuming a soft tissue density of 1 g/cm^3^. Target-to-background ratios (tumor_max_/heart_mean_, tumor_max_/muscle_mean_, spleen_max_/heart_mean_, spleen_max_/muscle_mean_) were calculated to correct for background levels. Mice in the XRT + anti-CTLA-4 group were stratified into two groups, CD8a low (< 6) and CD8a high (> 6), based on the tumor-to-heart ratio (*N* = 5/group). The median tumor-to-heart ratio of the treatment group was applied as cutoff value.

### *Ex Vivo* Biodistribution

Mice undergoing longitudinal imaging (*N* = 4) underwent conventional *ex vivo* biodistribution analysis after the last imaging session. The axillary lymph node (ALN), cervical lymph node (CLN), inguinal lymph node (ILN), blood, heart, lungs, liver, kidneys, stomach, intestine, muscle, bone, brain, thymus, and tumor were resected, weighed, and the radioactivity counted in a gamma counter (Wizard^2^, PerkinElmer).

### Immunohistochemistry

Paraffin-embedded tumors and spleens were sectioned at 4 μm, slides deparaffinized in Histo-Clear II (#12358637, Fisher Scientific) and rehydrated in a series of alcohols prior to heat-induced epitope retrieval in citrate buffer pH = 6. Sections were blocked with Peroxidase Blocking Solution (#S2023, Agilent Technologies) for 10 min and 2 % bovine serum albumin (BSA) in PBS for 20 min followed by incubation with recombinant anti-mouse CD8a^+^ antibody raised in rabbit (#ab209775, Abcam) at 1:1000 dilution for 1 h at RT. Primary antibody was detected using the EnVision+ System-HRP Labeled Polymer and Liquid DAB+ Substrate Chromogen System (Agilent Technologies) for 40 min and sections were counterstained with Mayer’s Hematoxylin (Region H Apotek). All procedures were performed at room temperature and all slides were stained in the same analysis.

### Flow Cytometry

Tumors were chopped and processed into single cell suspensions using a mouse tumor dissociation kit (#130-096-730, Miltenyi Biotec) according to the manufacturers’ protocol (*N* = 6/group). Spleens were mashed with a plunger through a 70-μm cell strainer (*N* = 6/group). Cells were washed twice in FACS buffer (PBS without Ca^2+^ and Mg^2+^, 1 % BSA, 0.5 mM EDTA, 0.1 % NaN_3_) and approximately ten million cells pre-incubated with Fc block (#553142, BD Biosciences) for 15 min. Cells were washed and stained for viability and cell surface markers according to standard procedures. The following mouse antibodies were used: anti-CD8a (BB515, clone 53-6.7, # 564422, BD Biosciences), anti-CD45 (AF700, clone 30-F11, #560510, BD Biosciences), and anti-CD3 (AF700, clone 145-2C11, #100308, BioLegend). Cells were acquired on a LSRFortessa flow cytometer (BD Biosciences). Data were collected using BD FACSDiva Software (v1.6) and further analyzed with FlowJo v10.4.2 (Tree Star Inc.).

### Statistical Analyses

**Fig. 1.**
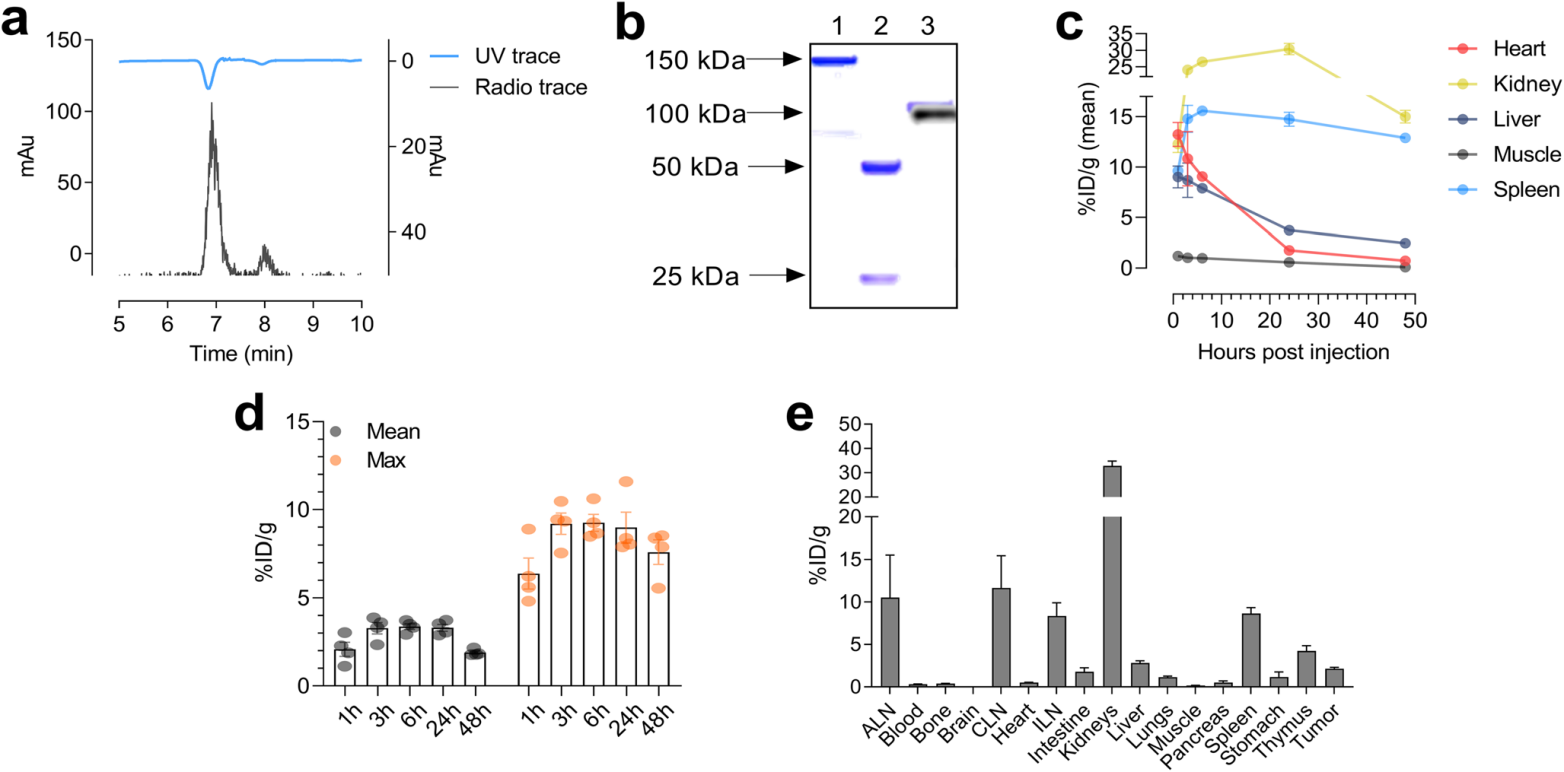
**a** Representative HPLC chromatogram of [^64^Cu]NOTA-CD8a. **b** Overlay of Coomassie staining and radiography of SDS-PAGE gel. Lane 1, full length CD8a^+^ antibody; lane 2, reduced full length CD8a^+^ antibody; lane 3, [^64^Cu]NOTA-CD8a. **c***In vivo* biodistribution of [^64^Cu]NOTA-CD8a in major organs derived from PET ROI analysis of the heart, kidney, liver, muscle, and spleen and expressed as % ID/g at 1, 3, 6, 24, and 48 h p.i*.* (*N* = 4). **d** Mean and maximum uptake of [^64^Cu]NOTA-CD8a in tumors derived from PET ROI analysis and expressed as % ID/g (*N* = 4). **e***Ex vivo* biodistribution after the last imaging session 48 h p.i. of [^64^Cu]NOTA-CD8a derived from gamma counting of tissues and expressed as %ID/g (*N* = 4). Data are presented as mean ± SEM. ALN, axillary lymph node; ILN, inguinal lymph node; CLN, cervical lymph node; % ID/g, % injected dose per gram tissue.

**Fig. 2.**
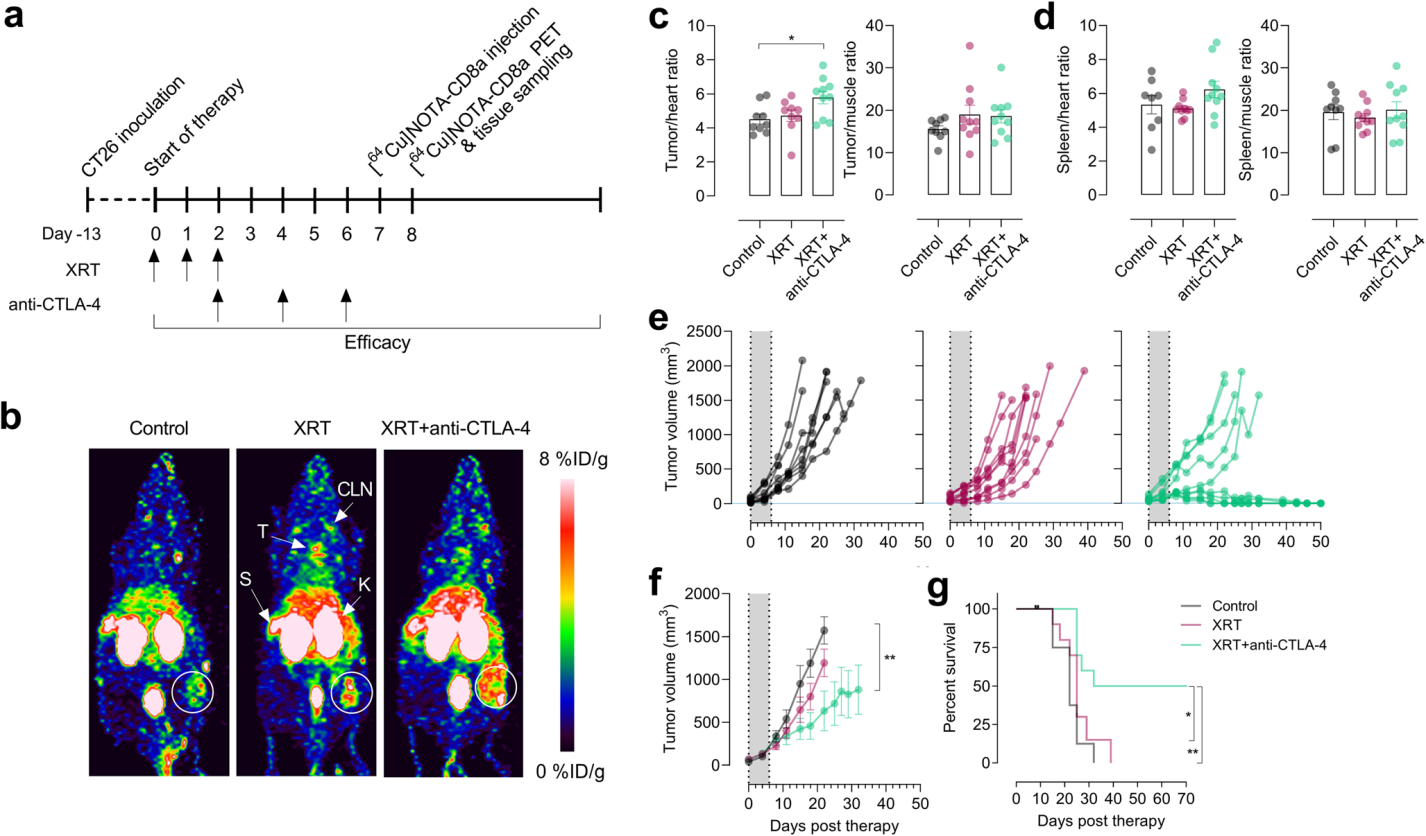
**a** Overview of timing of model establishment, therapy regimen, and [^64^Cu]NOTA-CD8a injection and scanning (*N* = 16/group). Radiation therapy (XRT) was performed as fractionated doses for three consecutive days (3 × 2 Gy) and anti-mouse CTLA-4 antibody was dosed i.p. at 10 mg/kg three times. **b** Representative maximum intensity projection PET images of a mouse from each treatment group at 24 h p.i. of [^64^Cu]NOTA-CD8a illustrating uptake in thymus (T), lymph nodes (cervical lymph node, CLN), spleen (S), kidneys (K), and tumors (designated by circle). **c** Tumor-to-heart and tumor-to-muscle ratios of [^64^Cu]NOTA-CD8a. **d** Spleen-to-heart and spleen-to-muscle ratios of [^64^Cu]NOTA-CD8a. **e** Tumor growth of individual mice in each treatment group from the time of randomization (day 0) until 1500 mm^3^ or end of study. The gray area represents the treatment period. **f** Mean tumor growth from the time of randomization (day 0) until > 50 % of mice in each treatment group was euthanized. The gray area represents the treatment period. **g** Survival proportions of mice in the different treatment groups. All uptake values are derived from PET ROI analysis and expressed as % ID/g. Data are presented as mean ± SEM (*N* = 10/group for all graphs). The significance level is indicated by asterisks (*). *p < 0.05, **p < 0.01, ***p < 0.001, ****p < 0.0001. XRT, external radiation therapy; % ID/g, % injected dose per gram tissue.

**Fig. 3.**
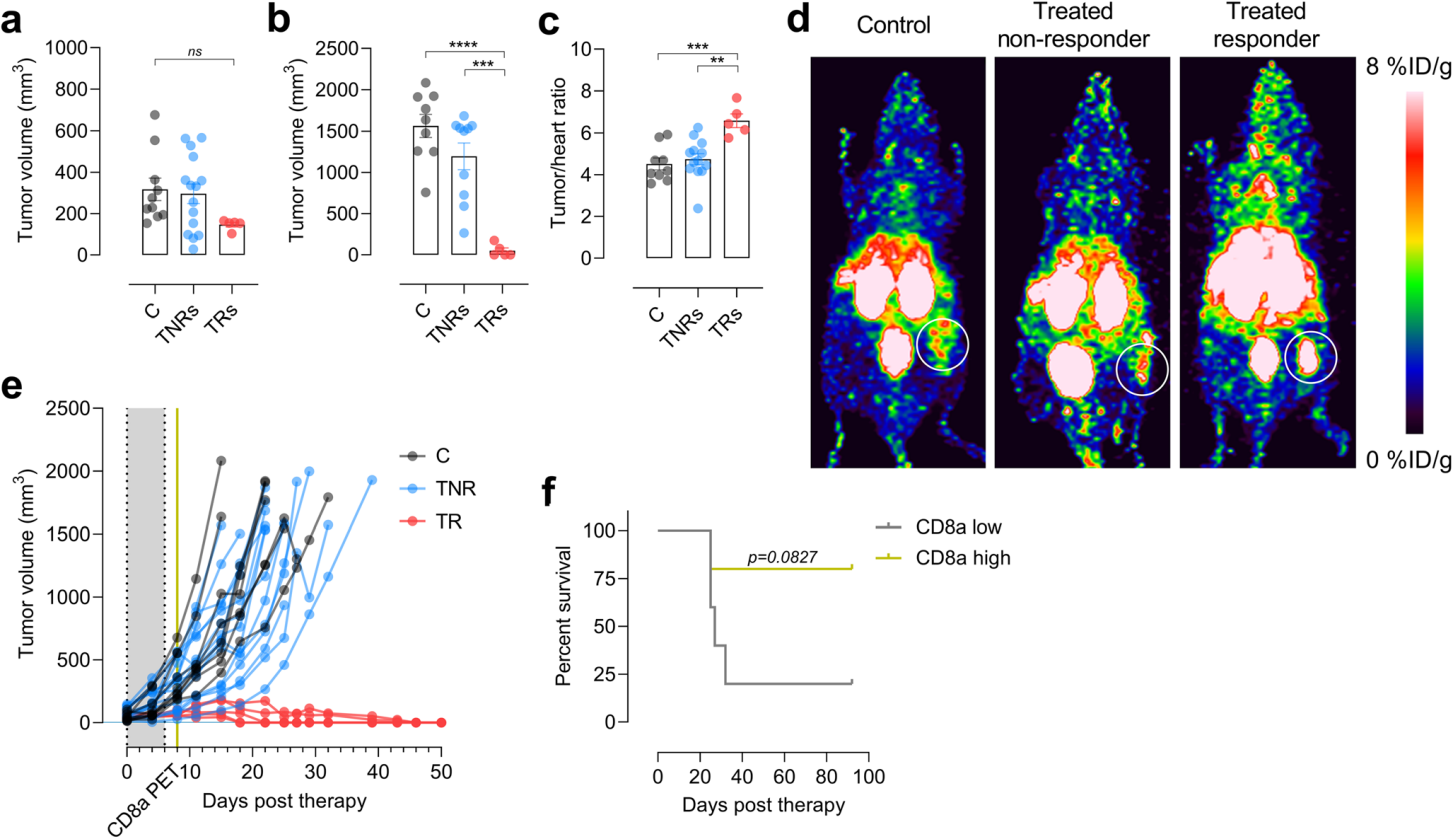
Tumor volume of mice on **a** the day of [^64^Cu]NOTA-CD8a PET imaging (day 8) and **b** day 22 grouped by control (black, *N* = 10), treated non-responders (blue, *N* = 15), and responders to therapy (red, *N* = 5). **c** [^64^Cu]NOTA-CD8a PET tumor-to-heart ratio in control (*N* = 10), treated non-responders (*N* = 15), and treated responders (*N* = 5). **d** Representative maximum intensity projection PET images of a control, treated non-responder, and a treated responder. Circles designate the tumor. **e** Tumor growth curves from day 0 until 1500 mm^3^ or end of study. The gray area represents the treatment period and the yellow line the time of [^64^Cu]NOTA-CD8a PET imaging. **f** Survival proportions of mice stratified on low (< 6) or high (> 6) [^64^Cu]NOTA-CD8a tumor-to-heart ratio in XRT + anti-CTLA-4-treated mice (*N* = 5/group). All uptake values are derived from PET ROI analysis and expressed as % ID/g. Data are presented as mean ± SEM. The significance level is indicated by asterisks (*). *p < 0.05, **p < 0.01, ***p < 0.001, ****p < 0.0001. C, control; TNR, treated non-responder; TR, treated responder.

Data are stated as mean ± SEM. One-way ANOVA with *post hoc* test corrected for multiple comparisons (Tukey) was applied to test for tumor volumes between groups (days 0, 4, 8, and 22), image contrast over the imaging time course (Fig. 1d), and the target-to-background ratios between treatment groups (Fig. 2c, d, Fig. 3c). Two-way ANOVA with repeated measures and Tukey’s multiple comparisons test was applied to compare tumor volumes over time until day 22 (Fig. 2f) and target-to-background ratios over the imaging time course (Table 1). Survival was analyzed using the Kaplan-Meier method and the Log-rank (Mantel-Cox) test, where *p* < 0.017 was considered statistically significant when correcting for multiple comparisons using the Bonferroni method (Fig. 2g). *p* values ≤ 0.05 were considered statistically significant. Statistical analyses were performed using GraphPad Prism 8.0c (GraphPad Software).

**Table 1.** Target-to-background ratios of [^64^Cu]NOTA-CD8a derived from ROI analysis of PET/CT images in CT26 tumor-bearing mice (*N* = 4/timepoint)

	Tumor/heart	Tumor/muscle	Spleen/heart	Spleen/muscle
1 h	0.15 ± 0.02	2.13 ± 0.46	0.93 ± 0.14	14.03 ± 3.52
3 h	0.24 ± 0.02	2.82 ± 0.32	1.17 ± 0.05	13.87 ± 1.04
6 h	0.35 ± 0.01	3.43 ± 0.58	1.55 ± 0.07	13.82 ± 2.57
24 h	1.89 ± 0.28	5.92 ± 0.67	5.59 ± 0.46	17.14 ± 0.77
48 h	2.64 ± 0.09	7.89 ± 0.44	8.16 ± 0.32	36.62 ± 7.57

Values are mean ± SEM

## Results

### Radiolabeling and Longitudinal PET/CT Imaging

Rat-anti-mouse-CD8a was successfully conjugated to the NOTA chelator and DOL was determined to 4.0 NOTA per CD8a-F(ab)′2. NOTA-CD8a-F(ab)′2 was subsequently radiolabeled with Cu-64 with a radiochemical yield of 74.9 ± 3.0 % and a specific activity of 209 ± 34.2 MBq/μmol. The radiochemical purity was > 99 % and aggregates estimated to < 5 % by SEC-HPLC. A representative HPLC chromatogram of [^64^Cu]NOTA-CD8a is shown in Fig. 1a, where the UV peak corresponds to F(ab)′2. The major radioactive peak aligned with the F(ab)′2 UV peak. SDS-PAGE analysis and radiography confirmed the digestion efficiency and revealed a pure final product at 100 kDa (lane 3) consistent with the radioactive overlay (Fig. 1b).

The temporal *in vivo* distribution of [^64^Cu]NOTA-CD8a was assessed by longitudinal PET/CT imaging in CT26 tumor-bearing mice 1, 3, 6, 24, and 48 h post-injection (p.i.). The temporal biodistribution for major organs is depicted in Fig. 1c and revealed high accumulation in the kidneys, spleen, liver, and heart at the initial imaging timepoints. [^64^Cu]NOTA-CD8a uptake decreased in the heart, kidneys, and liver at the late timepoints confirming clearance primarily through the kidneys and the hepatobiliary system. Quantitative data on the temporal uptake in tumors are depicted in Fig. 1d and the average tumor uptake was 2.08 ± 0.39, 3.28 ± 0.33, 3.37 ± 0.17, 3.30 ± 0.19, and 1.88 ± 0.09 %ID/g for the 1, 3, 6, 24, and 48 h timepoint, respectively. Likewise, the maximum uptake within tumors was 6.39 ± 0.88, 9.2 ± 0.60, 9.25 ± 0.48, 8.98 ± 0.89, and 7.59 ± 0.69 %ID/g for the 1, 3, 6, 24, and 48 h timepoint, respectively. Target-to-background ratios overall significantly increased throughout the imaging time course (*p* < 0.05, Table 1). *Ex vivo* biodistribution after the last imaging timepoint confirmed the PET data obtained with accumulation primarily seen in kidneys, lymphoid tissue, and tumor (Fig. 1e).

### Treatment Monitoring

The ability of [^64^Cu]NOTA-CD8a to detect differences in treatment-induced changes in CD8a^+^ cells was assessed in an immunotherapy combination study with XRT and a T cell engaging immune checkpoint inhibitor, anti-CTLA-4 (Fig. 2a). Mice were subjected to XRT alone or in combination with murine anti-CTLA-4 and underwent [^64^Cu]NOTA-CD8a PET/CT after the treatment regimen. Although highest target-to-background ratios were observed 48 h. p.i. in the longitudinal imaging study, 24 h p.i. was chosen for imaging with the rapid tumor growth of CT26 tumors in mind.

Representative maximum intensity projection PET images of a mouse from each treatment group are shown in Fig. 2b, where [^64^Cu]NOTA-CD8a primarily targeted lymphoid tissue and tumors. [^64^Cu]NOTA-CD8a PET uptake was corrected for background levels by dividing with the ROI-based uptake in the heart and muscle as a measure for specific signal in the target organs. The maximum tumor-to-background (tumor/heart, tumor/muscle) and spleen-to-background (spleen/heart, spleen/muscle) ratios of each treatment group are depicted in Fig. 2c and Fig. 2d, respectively. The tumor-to-heart ratio was found to be increased in mice receiving XRT and anti-CTLA-4 therapy (*p* = 0.0246), but no difference was found in the tumor-to-muscle, spleen-to-heart, or spleen-to-muscle ratios among treatment groups.

Tumor growth of individual mice within treatment groups showed a heterogeneous response to therapy (Fig. 2e) and was effectively inhibited in mice subjected to combined XRT and anti-CTLA-4 therapy compared to control (*p = 0.0025*) on day 22 (the last day of the control group), Fig. 2f. No effect of XRT alone compared to control was observed (*p* = 0.2021), nor was there a difference between XRT alone and XRT + anti-CTLA-4 (*p* = 0.1019). Correspondingly, median survival was 22, 25, and 62 days for the control, XRT, and XRT + anti-CTLA-4 groups, respectively. Overall survival was significantly improved in the XRT + anti-CTLA-4-treated mice compared with the control (*p* = 0.0017) and XRT-treated (*p* = 0.0119) mice (Fig. 2g).

### Response Prediction

As evident from the tumor growth curves in Fig. 2e, two distinct groups of responding and non-responding mice among the XRT + anti-CTLA-4-treated mice were observed. Based on this observation, all mice were retrospectively divided into groups of control, treated non-responders (TNRs), and treated responders (TRs) to investigate the accuracy of [^64^Cu]NOTA-CD8a PET for therapy monitoring and response prediction. TRs were defined as mice with no tumor regrowth for the entire study period of 92 days.

Tumor volumes of control, TNRs, and TRs had not diverged significantly yet at the time of imaging on day 8 (Fig. 3a) although a tendency was observed (*p* = 0.1629). On day 22 or at endpoint, the tumor volumes differed significantly, where the mean tumor volume of TRs was reduced compared to that of TNRs (*p* < 0.0001) and control (*p* = 0.0002) mice (Fig. 3b). Based on the observed increase in [^64^Cu]NOTA-CD8a tumor-to-heart ratio of the XRT + anti-CTLA-4 group, the [^64^Cu]NOTA-CD8a tumor-to-heart ratio was plotted for control, TNRs, and TRs (Fig. 3c). The [^64^Cu]NOTA-CD8a tumor-to-heart ratio was significantly elevated in TRs compared to TNRs (*p* = 0.0018) and control (*p* = 0.0010) on day 8. There was no difference in uptake between control and TNRs (*p* = 0.8232). The differential uptake pattern among responders and non-responders was also evident from the representative maximum intensity projection PET images (Fig. 3d). Tumor growth curves of all mice now grouped as control, TNRs, and TRs further confirmed the inhibited tumor growth in the high [^64^Cu]NOTA-CD8a uptake group (Fig. 3e).

To further investigate the sensitivity of [^64^Cu]NOTA-CD8a PET for prediction of response, XRT + anti-CTLA-4-treated mice were stratified into two groups based on the tumor-to-heart ratio. The tumor-to-heart ratio of mice in the XRT + anti-CTLA-4 group ranged between 4.15 and 7.66 with a median of 6. The median tumor-to-heart ratio was applied to stratify mice into either CD8a low ([^64^Cu]NOTA-CD8a < 6) or CD8a high ([^64^Cu]NOTA-CD8a > 6) uptake. Overall survival was not improved in the CD8a high group (*N* = 5) compared with the CD8a low group (*N* = 5) (*p* = 0.0827, Fig. 3f).

### Therapy Induced Changes in CD8a^+^ Populations Detected with [^64^Cu]NOTA-CD8a

To validate the findings obtained with [^64^Cu]NOTA-CD8a PET, the treatment regimen was applied to a new set of mice for tissue isolation and *ex vivo* analysis of CD8a^+^ numbers. Tumors and spleens were isolated from mice on day 8 after initiation of therapy (Fig. 2a) and subjected to flow cytometric and immunohistochemical analyses.

**Fig. 4.**
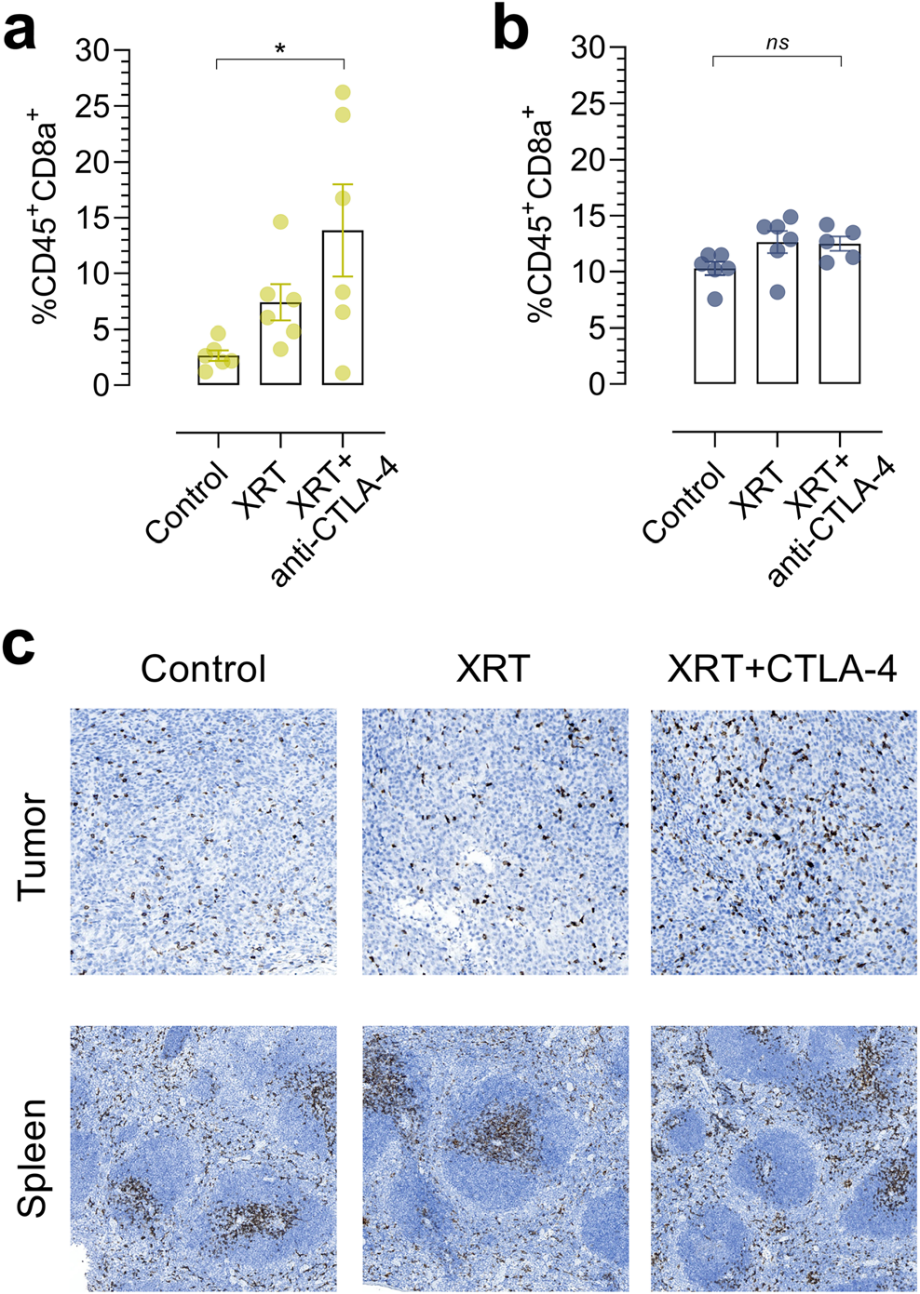
Flow cytometric analysis of **a** tumors and **b** spleens harvested from control, XRT-, and XRT + anti-CTLA-4-treated mice on day 8 following treatment initiation (*N* = 6/group for tumor, *N* = 5–6/group for spleen). **c** Representative immunohistochemical staining of CD8a^+^ in paraffin-embedded tumors and spleens harvested from control, XRT-, and XRT + anti-CTLA-4-treated mice on day 8 following treatment initiation. Data are presented as mean ± SEM. The significance level is indicated by asterisks (*). *p < 0.05, **p < 0.01, ***p < 0.001, ****p < 0.0001. XRT, external radiation therapy.

Flow cytometric analysis showed an approximate 5-fold increase in %CD45^+^CD8a^+^ cells in tumors of XRT + anti-CTLA-4-treated mice that was significantly higher than that of the control group (*p* = 0.0204) (Fig. 4a). No difference in tumor-infiltrating CD45^+^CD8a^+^ cells was found between the XRT and control (*p* = 0.4168) nor the XRT- and XRT + anti-CTLA-4 (*p* = 0.2157)-treated groups. A similar pattern could be detected in spleens of XRT + anti-CTLA-4-treated mice, where control, XRT-, and XRT + anti-CTLA-4-treated mice presented with 10.29 ± 0.6, 12.64 ± 0.9, and 12.49 ± 0.6 %CD45^+^CD8a^+^ cells, respectively (Fig. 4b). The groups did not differ significantly, however (*p* = 0.0837).

Immunohistochemical analysis of tumors and spleens confirmed this variable CD8a^+^ infiltration among different treatment groups (Fig. 4c). Overall, the flow cytometric and immunohistochemical analyses supported the uptake pattern of [^64^Cu]NOTA-CD8a in CT26 tumor-bearing mice subjected to XRT alone or in combination with anti-CTLA-4 therapy.

## Discussion

The molecular and cellular features associated with a successful immune response to a tumor are continuously investigated and mapped to identify key denominators of response to immunotherapy. One of the major challenges is the effective stratification of responders from non-responders in the clinical setting. While immune monitoring of patients with serial biopsies or peripheral blood samples is extremely informative, the location of sampling, *i.e.*, tumor heterogeneity or organ-dependent differences, timing of sampling, and interpretation of data before and after therapy are all potential contributors to biological variation that complicate the examination of data [22]. Therefore, we developed a specific radiotracer to be used for non-invasive detection, tracking, and quantification of CD8a^+^ lymphocytes in mice with the possibility of repeated imaging and spatial mapping over time.

F(ab)′2 fragments of a CD8a^+^ antibody were successfully produced and concurrently purified by preparative HPLC. Importantly, there were no full length CD8a^+^ antibodies or Fc fragments in the final product as confirmed by SDS-PAGE. Intact monoclonal antibodies targeting lymphocyte surface markers can have substantial immunomodulatory effects and can deplete the target cell population *in vivo* limiting their utility as imaging agents [23, 24]. The purified CD8a^+^-F(ab)′2 fragments were radiolabeled with Cu-64 with a high radiochemical purity. Many immuno-PET tracers have been coupled to Zirconium-89 (Zr-89, *t*_1/2_ = 78.4 h) due to the relatively long biological half-lives of antibodies. The use of Cu-64, however, reduces radiation exposure and allows for multiple scanning sessions to be conducted in the same animal. Furthermore, the shorter positron range of Cu-64 compared with Zr-89 (0.7 *vs.* 1.18 mm) enables more exact quantification of uptake in smaller organs such as lymph nodes.

Our *in vivo* studies demonstrated that [^64^Cu]NOTA-CD8a targeted lymphoid tissue, *i.e.*, the spleen, lymph node, and the thymus of immunocompetent mice. The optimal dose of CD8a^+^-F(ab)′2 for tumor visualization has previously been established in immunocompetent mice by our group [25]. The optimal dose was applied in this study to block endogenous CD8a^+^ levels and thereby increase the amount of tracer circulating in the blood. *In vivo* biodistribution identified the optimal imaging timepoint as 24 h after injection of tracer, where the mean [^64^Cu]NOTA-CD8a tumor uptake peaked. Although an increase in image contrast was observed from 24 to 48 h p.i. (Table 1), the fast tumor growth of the CT26 model was the rationale for selecting 24 h for evaluation of treatment efficacy. This was based on the fact that the magnitude of non-specific uptake of antibodies increases with tumor size due to the enhanced permeability and retention of macromolecules [26, 27]. Thus, the potential differences in tumor volume between different treatment groups could possibly be more pronounced 48 h as opposed to 24 h p.i. thus introducing a bias in interpretation of the imaging data. Moreover, tumors that are prominently different in size does not eliminate the possibility that the observed differences in CD8a^+^ infiltrate could be a consequence of tumor growth rather than a response to treatment. Importantly, tumor volumes were not significantly different at the day of PET imaging in this study. At 24 h p.i., approximately 2 %ID/g tracer remained in the circulation. Therefore, target uptake was corrected for background levels by normalizing to the heart content of tracer. In this way, tumoral and splenic uptake was corrected for potential treatment-induced changes in perfusion thereby reflecting residual CD8a^+^ numbers.

Other CD8a^+^-targeting antibody-derived probes have shown similar tumor uptake in syngeneic mouse tumor models. The [^89^Zr]malDFO-169 cDb (~ 55 kDa) [16] presented with a PET-derived tumor-to-blood ratio of ~ 2 at 24 h p.i. in CT26 tumor-bearing mice, which is in good agreement with the tumor-to-heart ratio of 1.89 ± 0.28 obtained in this study. In contrast, the [^89^Zr] PEGylated VHH-X118 camelid (~ 15 kDa) [18] exhibited a tumor-to-muscle ratio of ~ 10–20 at 24 h p.i. in B16F10 and Panc02 tumors. Typically, lower molecular weight corresponds to lower bioavailability and thereby amount of tracer available for tumor accumulation. Contrary, improved tumor penetration and the faster clearance from the blood stream increases image contrast at earlier timepoints. This, together with the fact that the authors utilized different tumor models likely explains the superior tumor-to-muscle contrast of [^89^Zr] PEGylated VHH-X118.

Combining conventional anti-cancer therapies with immune checkpoint inhibitors is currently investigated in numerous clinical trials to improve patient benefit [28]. Especially, the synergy of immunogenic cell death induced by irradiation leading to local and systemic immune responses in combination with immunotherapy has gained much attention [29]. Both the local and systemic CD8a^+^ infiltration was investigated with [^64^Cu]NOTA-CD8a in this study, *i.e.*, splenic and tumoral uptake (Fig. 2c, d). In tumors treated with radiotherapy in combination with anti-CTLA-4 therapy, a significantly increased tumor-to-heart ratio was observed. We found no effect of XRT or XRT + anti-CTLA-4 on the [^64^Cu]NOTA-CD8a spleen-to-heart or spleen-to-muscle ratio suggesting that our probe was not able to detect splenic changes or that our dosing regimen was not sufficient in producing systemic effects of focal radiation. Sparse preclinical data exist on the abscopal effect of radiation therapy on splenic CD8^+^ levels in combination with immune checkpoint inhibitors. Increased CD8^+^ numbers in the spleen of mice have previously been demonstrated on day 8 following a fractionated 3 × 9.18 Gy dose in combination with anti-PD-1 [30]. The exact T cell infiltration kinetics indeed depend on murine tumor model and XRT dose schedule [31] and it is plausible that our dosing regimen or the timing of PET imaging was not optimal to detect systemic changes in CD8a^+^ numbers. This notion is supported by the flow cytometric *ex vivo* analysis, which did not show differences in splenic CD45^+^CD8a^+^ numbers among treatment groups on day 8 following therapy initiation (Fig. 4b). Further, the increase in the [^64^Cu]NOTA-CD8a tumor-to-heart ratio in tumors of mice treated with XRT and anti-CTLA-4 matched the increased number of CD45^+^CD8a^+^ cells detected by flow cytometry in this treatment group. Overall, the *ex vivo* evaluation of tissue confirmed the impact of our efficacy protocol and the specificity as well as accuracy of our CD8a^+^ PET imaging probe for monitoring treatment-induced responses in CD8a^+^ dynamics in this particular setup.

In tumors treated with combined XRT and immune checkpoint inhibition of CTLA-4, we identified TRs and TNRs. There was no pronounced effect of treatment on tumor volumes between groups at the time of imaging. Interestingly, TRs presented with an increased [^64^Cu]NOTA-CD8a tumor-to-heart ratio compared with TNRs. This indicates that the utility of [^64^Cu]NOTA-CD8a could extend beyond monitoring and might be a useful tool for predicting response to therapy. However, the arbitrary cutoff value (median [^64^Cu]NOTA-CD8a tumor-to-heart ratio of XRT + anti-CTLA-4-treated mice) used for stratification was not an indicator of overall survival.

The utility of CD8^+^ as a predictive and prognostic biomarker of response has been lively debated. The main question arising is that the presence of CD8^+^ cells in the tumor microenvironment is not a prerequisite for response to immunotherapy, as the CD8^+^ cells could be anergic and/or exhausted and not capable of promoting tumor-mediated killing. Indeed, the activation state and clonality of T cells have been proposed as critical determinants of treatment response to immunotherapy [32, 33]. To address this issue, PET tracers targeting activation markers of T cells or soluble proteins such as cytokines are increasingly being developed [23, 34, 35]. Although providing significant information, this approach might be limited since activation markers can be short-lived following an immunotherapeutic treatment regimen necessitating a well-timed scanning schedule for accurate prediction or multiple scanning sessions—limiting its clinical utility. In addition, targeting secreted markers such as cytokines might face issues with solubility and redistribution within the tissue.

Although our data suggest that [^64^Cu]NOTA-CD8a could be a suitable *in vivo* biomarker for response prediction and monitoring of immunotherapy protocols, additional experiments are warranted including different combination strategies, tumor models as well as extending to orthotopic models, as the immune infiltrate and immunotherapeutic efficacy are known to differ with implantation site [36]. Also, it would be interesting to apply the [^64^Cu]NOTA-CD8a criterion dictating response in this study to a new cohort of mice to confirm the accuracy and reproducibility in determining response.

## Conclusions

In the present study, we developed a Cu-64 labeled antibody-based PET tracer for the non-invasive detection and quantification of murine CD8a^+^ cells. We show that [^64^Cu]NOTA-CD8a can be used to detect and quantify CD8a^+^ cells and monitor response to a common immunotherapy combination protocol. Further, our data suggest that [^64^Cu]NOTA-CD8a PET may serve as a predictive imaging biomarker of response. Future work including additional preclinical examination across multiple cancer types and treatment regimens will elucidate whether [^64^Cu]NOTA-CD8a is feasible for clinical translation.
